# How does a simulated high-intensity functional training competition influence on stress, recovery and muscle power?

**DOI:** 10.3389/fspor.2025.1734355

**Published:** 2026-01-05

**Authors:** Paulo Vitor de Souza Barboza, Stephany Fernandes da Rocha Rodrigues, Rodolfo André Dellagrana, Alberto Kruschewsky, Déborah de Araújo Farias, Mateus Rossato

**Affiliations:** 1 Faculty of Physical Education and Physiotherapy, Federal University of Amazonas, Manaus, Brazil; 2 Department of Physical Education, State University of Ponta Grossa, Ponta Grossa, Brazil; 3 Faculty of Physical Education, State University of Santa Cruz, Ilhéus, Brazil

**Keywords:** fatigue, neuromuscular status, simulated competition, questionnaires, muscle recovery

## Abstract

High-intensity functional training (HIFT) has increased the number of practitioners who find in the modality a way to improve their health levels. HIFT sessions are called “Workout of the day” (WODs), and in addition to training sessions, some practitioners choose to participate in competitions (HIFT_C). Competitions are held on consecutive days and can raise stress levels and affect recovery. The aim of study was to evaluate the effects of a simulated HIFT_C on markers of stress, recovery, and muscle power. Fifteen men with experience in HIFT_C, participated. The HIFT_C was held over 2 days, 2 WODs on each day, and 2 h between WODs. Levels of general stress, and physical and emotional recovery were assessed using the RESTQ-Sport Questionary 48 h before and 24 h after the HIFT_C. The muscle power in the lower and upper limbs were assessed through the height of the Countermoviment Jump (CMJ) and medicine ball throw distance (AH), respectively. The results showed increases in somatic complaints, a reduction in success, and a reduction in sleep quality. In power parameters, when compared with baseline, we observed increase in AH in Pre-WOD 2, Post-WOD 3, Pre-WOD 4 and Post-WOD 4. We conclude that HIFT_C increases somatic complaints, reduces the perception of success, and impairs sleep quality and increases in power in the upper limbs.

## 1 Introduction

Regular physical activity provides benefits related to health promotion and quality of life for the practitioner. These benefits have contributed to the reduction in morbidity and mortality rates in the world population. Based on this knowledge, new training models have emerged and gained prominence, such as high-intensity functional training (HIFT). HIFT is characterized as a modality that emphasizes functional and multi-joint movements through strength and aerobic exercises (1). The training sessions are called “Workout of the day” (WOD) and are performed at higher intensities when compared to traditional aerobic or strength training sessions (2). Additionally, HIFT can be adapted to any level of experience of practitioners (3). Another characteristic of this modality is that the most experienced participants are periodically challenged to take part in competitions where their performances are tested.

Due to these characteristics, a series of studies have been carried out in recent years, with the aim of better understanding aspects related to the prevalence of injuries (4), psychological factors of practitioners (5, 6), nutritional interventions (7), fatigue (8), and recovery (9). Studies involving recovery have generally been dedicated to evaluating the effects after isolated training and training carried out during a single session (9, 10) or using models of real or simulated competitions (11, 12). We believed that competition-based models were particularly sensitive for detecting substantial disturbances in homeostasis. In addition to eliciting highly motivational and high-effort responses, these models were typically performed under conditions of incomplete recovery, as athletes often competed on two or three consecutive days. According to Zecchin et al. (12), two consecutive days of HIFT competition negatively affect the autonomic nervous system, but do not have a negative influence on muscle power and fatigue.

Furthermore, competitions promote metabolic, hormonal, and muscular performance disorders for up to 72 h after they take place (11). Therefore, monitoring parameters related to recovery after HIFT competitions it was necessary so that professionals involved in the modality could outline strategies that avoid the development of overreaching or injuries resulting from incomplete recovery. In this sense, professionals involved in HIFT training had increasingly sought sensitive metrics that were applicable to the real world, capable of providing information about the recovery status of their practitioners, whether after training or competitions.

Traditionally, studies that monitored fatigue and recovery after HIFT training or competitions have used blood markers (9, 11), markers of autonomic control (12), recovery perception scales (11), and the assessment of neuromuscular status through countermovement jumps (10, 12). However, the use of questionnaires, such as the RESTQ-Sport, appeared to be a low-cost and easy-to-apply alternative, capable of providing interesting information to coaches about the level of stress and recovery caused by training sessions or competitive periods (13–15). Although the RESTQ-Sport had been used in previous studies involving Crossfit® athletes (16, 17), to our knowledge, there was a gap in the literature regarding the use of this instrument to evaluated the effects of a simulated HIFT competition. Therefore, the present study aims to evaluate the effects of a simulated HIFT competition on stress, recovery, and muscle power.

## 2 Materials and methods

### 2.1 Study design

This investigation adopted a quasi-experimental, repeated-measures, short-term longitudinal design, conducted in an applied field setting. Following the framework proposed by (18) or professional practice in applied performance analysis, the study is classified as an Applied Performance Analysis – Quasi-Experimental Applied Intervention, given its emphasis on monitoring athlete responses within an ecologically valid and competition-like environment. All participants were exposed to the same simulated high-intensity functional training (HIFT) competition, which served as a controlled intervention. Performance, stress, and recovery markers were assessed at multiple time points (baseline, pre-WOD, post-WOD, and post-competition), enabling the examination of within-participant changes over time. No control group was included, consistent with quasi-experimental applied research commonly used to evaluate athlete responses in realistic training and competitive environments. The use of psychological and neuromuscular indicators aligns with the principles of applied performance analysis aimed at supporting decision-making in training and competition.

### 2.2 Participants

The study included 15 healthy men (31.5 ± 2.8 years; 83.5 ± 7.4 kg; 176 ± 9 cm) with more than 1 year of experience in the sport, a weekly training frequency of more than 3 days, and experience in HIFT competitions at regional level. All methodological procedures were approved by the University Ethics Committee, in accordance with the Declaration of Helsinki.

### 2.3 Experimental design

The study was conducted over a period of 8 days. Initially, participants performed 2 HIFT training sessions (Days 1 and 2). On Day 3 (baseline), in addition to the HIFT training, power markers of the lower limbs (CMJ) and upper limbs (medicine ball throw) were also assessed. On Days 4 and 5, there were no HIFT training sessions (Day-off), but on Day 4, participants answered the RESTQ-Sport questionnaire. The simulated HIFT competition was held on Days 6 and 7. On Day 8, athletes answered only the RESTQ-Sport questionnaire.

### 2.4 Assessment of stress and recovery levels

Stress and recovery levels were monitored using the RESTQ-Sport questionnaire (19), validated for the Portuguese language through reliability and test-retest evaluations (20). The questionnaire was sent via Google Forms to volunteers who responded on Day 4 (48 h before the simulated competition) and Day 8 (after the simulated competition). The RESTQ-Sport had been used to monitor stress and recovery levels (physical/mental) of athletes exposed to potentially stressful events, recovery phases, and their possible subjective consequences in the previous three days/nights. The questionnaire contains 19 multidimensional scales (1st order factor): general stress, emotional stress, social stress, conflicts/pressure, fatigue, lack of energy, somatic complaints, success, social recovery, physical recovery, general well-being, sleep quality, disruptions in breaks, emotional exhaustion, injuries, being in shape, self-acceptance, self-efficacy, and self-regulation. The 19 scales are divided into: 7 scales related to general stress, 3 to specific stress, 5 to general recovery, and 4 to specific recovery (2nd order factor). These scales reflected information about the athletes' emotional routines during training and their lives outside of the training and competition environment. Each participant indicated values from 0 to 6 on a Likert scale, where 0 indicated never having felt that feeling and 6 indicated always having felt that feeling in the previous 3 days/nights (14).

### 2.5 Muscular power assessment

Left-limb muscular power was assessed using the Elite Jump System (São Paulo, Brazil) jumping platform, using 3 countermovement jumps (CMJ). The highest jump was recorded, and the peak relative power (PRP) and peak absolute power (PAP) were calculated from the results. Upper-limb power was assessed by throwing a 20-lb medicine ball. Each participant threw 3 times, and the greatest distance reached during the throw was recorded. To perform the throw, the participants remained standing and supported with their backs against the wall. To be considered valid the throw, the anterior detachment of the trunk should not be observed. The measurement of the medicineball throw was considered the distance between the wall and the point of the first contact of the ball with the ground. Muscular power assessments were always preceded by a standard five-minute warm-up on the Assault Bike Classic. Post-WOD assessments were performed immediately after the WOD.

### 2.6 Subjective perception of effort

To assess the subjective perception of effort (RPE), the Borg CR10 scale adapted (21) was used after all WODs of the simulated competition, on a scale from 0 to 10, where 0 indicated minimum effort and 10 maximum effort.

### 2.7 Simulated competition

On Day 6, 2 WODs were performed, with a 2-hour interval between exercises. The same procedure was performed on Day 7. WOD 1 consisted of 5 rounds (400 m run + 30 box jumps with a box height of 60 cm + 30 wall balls – 20 lbs). WOD 2 was a “For time” consisting of 30 repetitions of Clean & Jerk – 135 lbs. WOD 3 was a “12-min Amrap”, 3 reps – 6 reps – 9 reps – 12 reps of the hang power snatch exercise −115 lbs + Toes to bar. WOD 4 was a 10 reps – 20 reps - 30reps consisting of front squat – 115 lbs + push press – 115 lbs + facing bar burpee. The choice of WODs, number of WODs per day, intervals between WODs, and order of execution took into account the need to cover different stimuli, such as aerobic and anaerobic resistance, and strength and power, for both the upper and lower limbs, and was similar to other studies that also used simulated competition models (12, 22–24). The exercises used in each WOD were unknown to the participants until the moment of the start of the simulated competition. This strategy was used to further increase psychological stress, characteristic of these competitions.

### 2.8 Statistical analysis

To assess changes in the parameters evaluated, the Cohen's (d) effect size (ES) was used. The ES was classified as <0.5 (small), 0.5–0.8 (moderate), and >0.8 (large). The choice of this approach was due to the need for a more practical and clear interpretation of the results. For the RESTQ-Sport questionnaire values, the ES was calculated from the means obtained at the moments before and after the simulated competition. For the power parameters at the different moments, the baseline values were used as the reference. The intraclass correlation coefficient (ICC) was used to analyze the reproducibility of the power parameters, and correlations were performed between the moments of baseline (Day 3) and Pre-WOD 1 (Day 6). All statistical analyses were performed in Windows Excel spreadsheets.

## 3 Results

Regarding the RPE of the WODs, mean values of 8 ± 1.35 (WOD 1), 7 ± 1.41 (WOD 2), 7 ± 1.13 (WOD 3), and 8 ± 1.26 (WOD 4) were observed. These values indicate that the intensities were between moderate and intense. The values for the 1st order factor of the RESTQ-Sport are presented in Table 1. Moderate ES values were observed for increases in somatic complaints (ES = 0.50; 28.5%), a reduction in success (ES = 0.50; 9.16%), and a reduction in sleep quality (ES = 0.52; 13.7%).

**Table 1:** Mean values, standard deviations, and effect sizes for the 1st order factor scales of the RESTQ-sport, assessed on Day 4 (48 h Pre) and Day 8 (24 h post).

N°	RESTQ-76 sport	Day 4 (48 h pre)	Day 8 (24 h post)	
	1st order factor	Mean ± SD	Mean ± SD	ES
1	General stress	1.07 ± 0.79	1.35 ± 1.18	0.29 (P)
2	Emotional stress	1.62 ± 0.65	1.78 ± 1.21	0.18 (P)
3	Social stress	1.08 ± 0.72	1.33 ± 1.23	0.26 (P)
4	Conflicts/Pressure	2.30 ± 0.91	2.65 ± 1.05	0.37 (P)
5	Fatigue	2.27 ± 1.34	2.50 ± 1.08	0.20 (P)
6	Lack of energy	1.53 ± 0.64	1.85 ± 0.98	0.40 (P)
7	Somatic complaints	**1.75 ± 1.16**	**2.25 ± 1.01**	**0** **.** **50 (M)**
8	Success	**3.60 ± 0.67**	**3.27 ± 0.73**	**0** **.** **50 (M)**
9	Social recovery	3.45 ± 1.16	3.68 ± 1.00	0.22 (P)
10	Physical recovery	3.52 ± 1.00	3.22 ± 0.90	0.33 (P)
11	General well-being	4.08 ± 0.70	4.05 ± 0.98	0.04 (P)
12	Sleep quality	**3.63 ± 0.72**	**3.13 ± 1.20**	**0** **.** **52 (M)**
13	Breakthrough disturbances	1.83 ± 1.39	2.43 ± 1.50	0.43 (P)
14	Emotional exhaustion	1.43 ± 1.16	2.03 ± 1.46	0.47 (P)
15	Injuries	3.12 ± 1.17	3.42 ± 1.35	0.25 (P)
16	Being fit	3.58 ± 1.26	3.35 ± 1.18	0.20 (P)
17	Self-acceptance	3.33 ± 1.02	3.32 ± 0.96	0.02 (P)
18	Self-efficacy	3.40 ± 1.09	3.10 ± 0.99	0.30 (P)
19	Self-regulation	3.17 ± 1.33	3.45 ± 1.04	0.25 (P)

Values in bold indicate a moderate effect size.

The 2nd order factor analyses of the RESTQ-Sport are presented in Table 2. Moderate effect sizes were found for increases in general stress (ES = 0.60; 18.0%) and specific stress (ES = 0.64; 23.4%), in addition to the reduction in general recovery (ES = 0.60; 5.19%).

**Table 2:** Mean values, standard deviations, and effect sizes for the 2nd order factor scales of the RESTQ-sport, assessed on Day 4 (48 h pre) and Day 8 (24 h post).

RESTQ-76 sport	Day 4 (48 h pre)	Day 8 (24 h post)	
2nd Order factor	Mean ± SD	Mean ± SD	ES
General stress	1.66 ± 0.50	1.96 ± 0.53	0.60 (M)
Specific stress	2.13 ± 0.88	2.63 ± 0.72	0.64 (M)
General recovery	3.66 ± 0.25	3.47 ± 0.39	0.60 (M)
Specific recovery	3.37 ± 0.17	3.31 ± 0.15	0.39 (P)

Table 3 presents the intraclass correlation values, confidence intervals, and determination factor obtained for the power parameters, evaluated at baseline (Day 3) and Pre-WOD 1 (Day 6). All variables showed strong correlation levels, indicating good reproducibility between measurements.

**Table 3:** Intraclass correlation coefficient (ICC), 95% confidence intervals, and determination factor for countermovement jump height (CMJ), peak anaerobic power (PPA), peak relative power (PRP), and medicine ball throw distance (AH).

Parameters	ICC [95% confidence intervals]	R^2^
CMJ (cm)	0.948 [0.847–0.983]	0.899
PPA (W)	0.970 [0.911–0.990]	0.942
PPR (W/kg)	0.975 [0.926–0.992]	0.952
AH (m)	0.741 [0.370–0.908]	0.550

**Figure 1:**
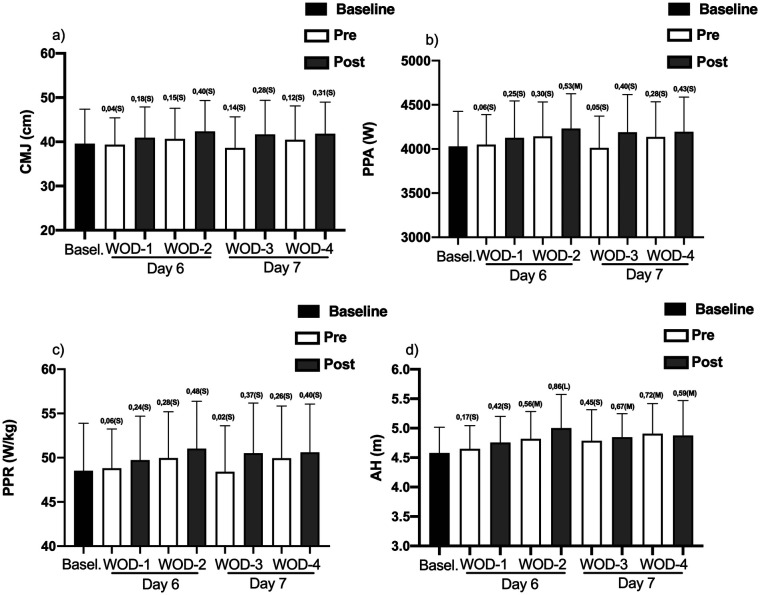
Power parameters [**(a)** CMJ, **(b)** PPA, **(c)** PPR and **(d)** AH] at different times [Baseline (black), Pre (white) and post (gray)] on Day 6 (WOD 1 and 2) and Day 7 (WOD 3 and 4) of the simulated HIFT competition. The effect size was calculated considering the baseline values, where S (small), M (moderate) and L (large).

Regarding the power parameters, the results are presented in Figure 1. Considering the effects of the simulated HIFT competition on height in the CMJ (Figure 1a), when compared with baseline, the results indicated small effect sizes for all moments evaluated. For the PPA (Figure 1b), when compared with the baseline results, a moderate increase in the effect size (ES = 0.53; 5.01%) was observed in Post-WOD 2. For the PPR, (Figure 1c) changes with a small effect size were observed for all moments evaluated. Regarding the AH (Figure 1d), when compared with the baseline values, we observed a moderate increase in Pre-WOD 2 (ES = 0.56; 5.18%), Post-WOD 3 (ES = 0.67; 5.57%), Pre-WOD 4 (ES = 0.72; 6.73%), and Post-WOD 4 (ES = 0.59; 6.1%). In addition, an elevation in the AH with a large effect size was observed at the Post-WOD 2 moment (ES = 0.86; 8.60%).

## 4 Discussion

The aim of the current study was to evaluate the effects of a simulated HIFT competition on general stress, recovery, and markers of muscular power. The hypothesis raised was that a simulated HIFT competition would cause worsening of markers of stress, recovery, and muscular power. Our findings partially confirmed our hypothesis, since a simulated HIFT competition was able to increase general and specific stress levels, in addition to decreasing general recovery. In contrast, a simulated HIFT competition caused increases in some parameters of muscular power, mainly in the upper limbs, without major changes in the lower limbs.

Monitoring the stress and recovery levels of training program participants is an important strategy for preventing overtraining. For this purpose, the RESTQ-Sport questionnaire, developed by (19), had been used by athletes from various sports (13–15). However, to the best of our knowledge, assessments of stress levels and recovery before and after a simulated HIFT competition have not yet been documented. D` Alpino et al. (17), evaluated the mood, stress, and recovery state among CrossFit® competitors and non-competitors. The authors demonstrated that CrossFit® practitioners subjected to periods of competition suffer changes in mood and stress levels when compared to non-competitive individuals subjected to the same training routines.

Our results indicated that a simulated HIFT competition led to an increase in general stress levels (ES = 0.60; 18.0%) and specific stress levels (ES = 0.64; 23.4%), in addition to a reduction in general recovery (ES = 0.60; 5.19%). Regarding general stress, all scales that compose this variable presented increases between the pre (Day 4) and post (Day 8) moments. However, only the somatic complaints scale showed a moderate effect size (ES = 0.50; 28.5%); this scale corresponds to physical discomfort and physical complaints related to the body as a whole. The increase in the scale for general stress was expected, and is in agreement with the study (25), since participants were exposed to a moderate to high intensity stimulus on consecutive days. Regarding specific stress (Scale 13–15), although small effect sizes were observed, the averages of the scales that compose this variable indicated a moderate effect size (ES = 0.64). For general recovery, with the exception of social recovery (Scale 9), all other scales showed reductions in scores after the simulated HIFT competition; however, scales 8 (success) and 12 (sleep quality) deserve to be highlighted, showing reductions in scores, with a moderate effect size (ES = 0.50; 9.16% and ES = 0.52; 13.7% respectively). The reduction in success (scale 8) would be associated with the feeling of not having been able to achieve the desired performance during the competition due to excessive self-demand (16). Regarding the reduction in sleep quality (scale 12), our findings are in agreement with the study by (13), who also reported worsening sleep quality during competitive periods. According to the author, changes in sleep quality may be indicative of a higher risk of injury. Another study that used the RESTQ to analyze stress and recovery was that of (16), who compared amateur Crossfit® athletes of both sexes (men vs. women) in a competition setting. The values of the general stress and general recovery scales are in agreement with our findings, with the values of these scales being higher among female volunteers compared to males.

Regarding the behavior of lower limb power, with the exception of the Post-WOD2 PPA, which presented a moderate effect size (ES = 0.53; 5.01%), all other parameters showed small effect sizes when compared to the baseline measurement (Day 3). These results indicate that a simulated HIFT competition, with the characteristics of our study, was not able to generate depreciation of physical valence. Our results corroborate the findings of (12). The authors simulated 2 days of Crossfit® competition (4 WODs) and evaluated power through CMJ before and after the 1st and 2nd days of competition. Although a reduction in CMJ height was observed, it was not significant. Although it is not considered a competition, benchmarking had been used as a common strategy to determine training parameters among practitioners. In this sense (10), evaluated, among other parameters, the recovery of muscular power (CMJ) after a Benchmark called Karen® (150 wall ball throws in the shortest time). The authors observed that 24 h after the Benchmark, CMJ values returned to the Pre-Benchmark values, indicating a rapid recovery. Furthermore (8), also performed CMJ assessments pre and post a Benchmark (Fran - 21 reps - 15 reps - 9 reps of Thrusters and Pull ups). The authors did not observe any negative effects on CMJ performance after 24 h.

Medicine ball throwing had been used to assess muscle power in several sports (26, 27), however, to the best of our knowledge, we pioneered the use of this method to monitor changes in anaerobic power both between WODs (pre and post) and between days (Day 6 and Day 7). Our results indicated that at different time points, throwing performance improved, with moderate to high effect sizes. We believe that these increases could be associated with physiological mechanisms related to the postactivation performance enhancement (PAPE) (28, 29). PAPE is considered a temporary increase in power performance after performing exercises with high overloads (30). According to (31), the PAPE can be explained by the mechanism of post-activation potentiation (PAP) involving the phosphorylation of the myosin light chain, which makes actin and myosin more sensitive to calcium, and, consequently, more likely to perform greater muscle contraction; or by the increase in the excitability of the alpha motor neuron (Hoffmann reflex) through strength exercises performed prior to power work. Noteworthy, the present study did not evaluate the PAPE mechanism; therefore, future research could benefit from analyzing PAPE responses during HIFT.

The PAPE has gained substantial interest in recent years, mainly because of the significant performance improvements reported in tasks that demand great muscular power production and the possibility that these can be applied by athletes to improve training efficiency and performance (32). For some researchers, the PAPE is used as a training method or as part of a warm-up routine, in preparation for high-intensity performance, an increase in strength would be expected when the muscle contraction in question was of short duration (33). The study carried out by (34) evaluated the PAPE effect between upper and lower limbs after performing an Olympic Clean & Jerk series with progressive loads (30%; 50%; 70%; and 90%) of 1RM, and the result obtained by the study showed potentiation of up to 12 min after the stimulus. This result corroborates our findings for AH, when we compared the moderate ES values for: baseline vs. pre WOD 2 (ES = 0.56; 5.18%), baseline vs. post WOD 3 (ES = 0.67; 5.57%), baseline vs. pre WOD 4 (ES = 0.72; 6.73%), and baseline vs. post WOD 4 (ES = 0.59; 6.1%), and the large ES value for: baseline vs. post WOD 2 (ES = 0.86; 8.60%).

Our study presents strengths and limitations. Among the limitations, the absence of blood markers, such as blood lactate and creatine kinase concentrations stands out. These variables could be indicators of physiological demands and muscle damage, respectively, and could provide information about the recovery process. Also, the fact that we did not include women, limited gender-dependent analyses. We evaluate a homogeneous sample of Brazilian HIFT practitioners, in which preclude the data generalization. Future studies involving other populations are encouraged. Although participants were instructed not to change their eating patterns, failure to control food intake, may have affected the results. Among the strengths of our study, we highlight the inclusion of tests to evaluate indicators of upper limb muscle power and the use of the RESTQ-Sport to evaluate markers of stress and recovery resulting from a simulated HIFT competition.

## 5 Conclusion

It can be concluded that a simulated HIFT competition is able to induce general and specific stress, in addition to impairing general recovery. Regarding the power parameters evaluated, the simulated HIFT competition did not cause major changes in lower limb power, but generated improvements in HA. Therefore, for better performance in competitive periods, coaches and athletes need to know and monitor the variables that can influence their performance, in order to minimize the losses caused on consecutive days of an HIFT competition, as well as to monitor pre-competition training and the tapering phase, so that the athlete starts the competition with their best performance.

## Data Availability

The raw data supporting the conclusions of this article will be made available by the authors, without undue reservation.
